# Concept of natural genome reconstruction.Part 5. Analysis of changes in the lifespan of old animals after reinfusion of bone marrow cells derived from old animals and treated with hDNAgr in combination with recombinant human angiogenin

**DOI:** 10.18699/vjgb-26-39

**Published:** 2026-05

**Authors:** V.S. Ruzanova, L.U. Grivtsova, S.G. Oshikhmina, A.S. Proskurina, G.S. Ritter, E.V. Dolgova, S.S. Kirikovich, E.V. Levites, Y.R. Efremov, T.D. Dubatolova, M.I. Meschaninova, A.L. Mamaev, O.S. Taranov, S.V. Sidorov, O.Y. Leplina, A.A. Ostanin, E.R. Chernykh, N.A. Kolchanov, A.S. Bryukhovetskiy, S.S. Bogachev

**Affiliations:** Institute of Cytology and Genetics of the Siberian Branch of the Russian Academy of Sciences, Novosibirsk, Russia; National Medical Research Radiological Centre of the Ministry of Health of the Russian Federation, Obninsk, Russia; Institute of Cytology and Genetics of the Siberian Branch of the Russian Academy of Sciences, Novosibirsk, Russia; Institute of Cytology and Genetics of the Siberian Branch of the Russian Academy of Sciences, Novosibirsk, Russia; Institute of Cytology and Genetics of the Siberian Branch of the Russian Academy of Sciences, Novosibirsk, Russia; Institute of Cytology and Genetics of the Siberian Branch of the Russian Academy of Sciences, Novosibirsk, Russia; Institute of Cytology and Genetics of the Siberian Branch of the Russian Academy of Sciences, Novosibirsk, Russia; Institute of Cytology and Genetics of the Siberian Branch of the Russian Academy of Sciences, Novosibirsk, Russia; Institute of Cytology and Genetics of the Siberian Branch of the Russian Academy of Sciences, Novosibirsk, Russia; Institute of Molecular and Cellular Biology of the Siberian Branch of the Russian Academy of Sciences, Novosibirsk, Russia; Institute of Chemical Biology and Fundamental Medicine of the Siberian Branch of the Russian Academy of Sciences, Novosibirsk, Russia; Laboratory Angiopharm LLC, Novosibirsk, Russia; State Scientific Center of Virology and Biotechnology “Vector” of Rospotrebnadzor, Koltsovo, Novosibirsk region, Russia; Novosibirsk State University, Novosibirsk, Russia City Clinical Hospital No. 1, Novosibirsk, Russia; Research Institute of Fundamental and Clinical Immunology, Novosibirsk, Russia; Research Institute of Fundamental and Clinical Immunology, Novosibirsk, Russia; Research Institute of Fundamental and Clinical Immunology, Novosibirsk, Russia; Institute of Cytology and Genetics of the Siberian Branch of the Russian Academy of Sciences, Novosibirsk, Russia; Clinical Hospital ‘‘NeuroVita’’, Moscow, Russia; Institute of Cytology and Genetics of the Siberian Branch of the Russian Academy of Sciences, Novosibirsk, Russia

**Keywords:** telomere length, life expectancy, double-stranded DNA preparation (hDNAgr), recombinant human angiogenin, pathomorphological analysis, reinfusion of bone marrow cells, длина теломеры, продолжительность жизни, препарат двуцепочечной ДНК (hDNAgr), ангиогенин рекомбинантный человеческий, патоморфологический анализ, реинфузия клеток костного мозга

## Abstract

Two series of tests were performed, on mice and rats, to assess the lifespan of old animals reinfused with bone marrow cells from old animals treated with fragmented human DNA (hDNAgr), recombinant human angiogenin, and both preparations together. Animals reinfused with untreated bone marrow cells from old animals or bone marrow cells from young animals were used as comparison groups. Using both outbred mice and CBA/Lac mice, no significant increase in the lifespan of animals reinfused with bone marrow cells treated with the hDNAgr was found compared with the group of mice reinfused with untreated bone marrow cells. Using the CBA/Lac line, mice reinfused with bone marrow cells treated with angiogenin simultaneously died of the characteristic symptom complex at 10 months after treatment. Pathomorphological analysis suggests that the simultaneous death of mice occurred as a result of pathological disorders in the excretory systems of animals. Reinfusion of bone marrow cells from old animals treated with angiogenin and hDNAgr and bone marrow cells taken from young animals significantly increases the lifespan of mice in groups. The combined use of two activators, angiogenin and hDNAgr, increased the average lifespan of 30 % of experimental mice to 35 months compared to 28 months in the control. Using Wistar rats as model animals in the first experiment, a reliable increase in the lifespan of rats with reinfusion of bone marrow cells from old animals treated with the hDNAgr preparation to 28 months was shown compared to the group that received untreated bone marrow cells from old animals, where the average lifespan of rats was 24 months. In the second similar experiment, no reliable difference in the lifespan of rats for the two groups was shown. Animals injected with bone marrow cells treated with angiogenin lived significantly longer than rats from the control group. The analysis of the amount of telomeric DNA in bone marrow cells of rats from the experimental and control groups 12 months after treatment showed that there was no significant increase in telomeric DNA. A molecular/cellular model of aging of the organism associated with the concept of “natural reconstruction of the genome” is considered.

## Abstract (ru)

Проведены две серии тестов (на мышах и крысах) по оценке продолжительности жизни старых животных, которым были реинфузированы клетки костного мозга старых животных, обработанные фрагментированной ДНК человека (hDNAgr), ангиогенином рекомбинантным человеческим и двумя препаратами совместно. В качестве групп сравнения использовались животные, которым реинфузировали необработанные клетки костного мозга старых животных или клетки костного мозга от молодых особей. В случае как аутбредных мышей, так и мышей линии CBA/Lac не выявлено достоверного увеличения продолжительности жизни животных при реинфузии клеток костного мозга, обработанных препаратом hDNAgr, по сравнению с группой мышей, которым реинфузировали необработанные клетки костного мозга. При использовании линии CBA/Lac мыши, которым реинфузировали клетки костного мозга, обработанные ангиогенином, на 10-й месяц после обработки одновременно пали от характерного симптомокомплекса. Патоморфологический анализ предполагает, что одновременная гибель мышей произошла в результате патологических нарушений в выделительных системах животных. Реинфузия клеток костного мозга от старых животных, обработанных ангиогенином и hDNAgr, и клеток костного мозга, взятых от молодых животных, значительно увеличивает продолжительность жизни мышей в группах. Совместное применение двух активаторов, ангиогенина и hDNAgr, увеличивало среднюю продолжительность жизни 30 % экспериментальных мышей до 35 мес. при 28 мес. в контроле. При использовании в качестве модельных животных крыс линии Вистар в первом эксперименте было показано достоверное увеличение жизни крыс при реинфузии клеток костного мозга старых животных, обработанных препаратом hDNAgr, до 28 мес. по сравнению с группой, получавшей необработанные клетки костного мозга старых животных, где средняя продолжительность жизни крыс составила 24 мес. Во втором аналогичном эксперименте достоверной разницы в продолжительности жизни крыс для указанных двух групп показано не было. Животные, которым вводили клетки костного мозга, обработанные ангиогенином, прожили достоверно дольше, чем крысы из контрольной группы. Проведенный анализ количества теломерной ДНК в клетках костного мозга крыс экспериментальных и контрольной групп через 12 мес. после обработки свидетельствовал, что достоверного увеличения теломерной ДНК не произошло. Рассматривается молекулярная/клеточная модель старения организма, связанная с концепцией «природной реконструкции генома».

## Introduction

The problem of aging is a dominant issue in modern biology, since the primary focus of scientific thought and applied research is searching for ways to halt the progressive increase in the number of people suffering from the so-called “diseases of civilization”, which are inherently related to this natural biological process.

Aging and death are fundamental, intrinsic biological properties representing the functioning and evolution of all living organisms, including humans. From a thermodynamic perspective, aging is a critical temporal threshold between the ability of an organism to reduce its entropy (also a fundamental intrinsic property of living systems) and successive progression toward an irreversible stationary state (i. e., death) characterized by maximum entropy (Lefever, 2018; Tlidi et al., 2018a, b). At the level of a living organism, aging is a dynamic pathophysiological process of accumulating alterations at multiple biological levels, leading to gradual decline in vital activity and, ultimately, death. The pace of this universal and irreversible process (within the framework of conventional existence) is governed by numerous external and internal factors. At its core, there lies “genetic aging” of the organism’s DNA repair machinery in the form of stem cells of various lineages that sustain coordinated responses maintaining internal homeostasis. All other pathological processes result from the failure of stem cells to preserve homeostasis (Bowen, Atwood, 2004; Mikheev et al., 2023).

The numerous existing theories of aging are generally categorized into two groups: the “programmed” (adaptive) and “damage” theories.

According to the programmed theories, functioning of a living organism is presumed to be biologically programmed by nature only for the period of its active life cycle, which includes development (i. e., organismal growth) and reproductive capacity (the so-called biological usefulness), determined by species-specific population expediency (da Costa et al., 2016). In other words, aging-related changes are regulated by a kind of biological sensor whose primary function is to monitor the developmental schedule of the organism until it reaches sexual maturity and reproductive capacity (Kozlov, 1999).

The damage theories posit that aging is not an inevitable sequela of an organism’s existence but rather results from accumulation of damage, or stochastic errors, in the genetic information storage and transmission systems. Over time, compensation for these impairments becomes infeasible, thus leading to death Kirkwood, 2005; Vijg, Campisi, 2008; Gems, Partridge, 2013; Mikheev et al., 2023)

Proponents of the programmed aging theories rely on the premise that aging is governed by genetic mechanisms orchestrating the evolution of living organisms. However, aging-related changes may also involve additional mechanisms not encoded by the genetic program, which exert non-programmed effects on the organism that also cause life termination. This effect may arise from stochastic cellular damage that alters the cell structure, function, and metabolism (and primarily, the membrane structure). This impact can also affect the genetic information as chromosomal DNA, which is believed to encode the aging program, thus potentially modulating its activation. Furthermore, toxic byproducts and free radicals are generated during normal cellular metabolic processes, their destructive activity being normally counterbalanced by cellular deactivation systems. Failure in these deactivation mechanisms, similar to external impacts, may cause damage to membranes and chromosomal DNA, which will also be accompanied by activation or modulation of aging mechanisms.Hence, both the approach conceptualizing aging as a genetically encoded evolutionarily programmed process (programmed aging) and the alternative paradigm viewing cellular damage as non-genetically determined yet either modulating or constituting the aging mechanism (nonprogrammed or nonadaptive aging) share fundamental biological commonality in explaining the causes of involution, senescence, and life termination.

Therefore, when analyzing organismal aging, we deem it more correct to shift focus from philosophical considerations of the reasons behind it to examining functional, cellular, and molecular-level alterations in the organism being indicators of aging (senescence markers). Each of these alterations accompanies aging and is a detectable manifestation of the ongoing involutionary process. We deliberately avoid using the term “aging mechanism”, since it means the initiation and development of events that are driven by the fundamental causes of senescence and currently remain beyond our scientific grasp.

There are many reviews classifying prominent detectable senescence markers. These reviews systematize the data characterizing indicators accompanying organism’s aging: genomic instability, telomere attrition, epigenetic modifications, loss of proteostasis, impaired macroautophagy, deregulated nutrient sensing, mitochondrial dysfunction, cellular senescence, stem cell exhaustion, altered intercellular communication, chronic inflammation, microbiota imbalance, and disrupted neuroendocrine regulation. Nearly all molecular and biochemical processes that can be classified according to specific feature and are affected by aging processes fall within these fundamental categories (Kenyon, 2010; López-Otín et al., 2013, 2023; Krauss, de Haan, 2016; Proshkina et al., 2020; Zhu et al., 2021; Mikheev et al., 2023).

One of the most remarkable findings of this study is the fact that extracellular double-stranded DNA (dsDNA) fragments delivered to hematopoietic stem cells (HSCs) via a natural biological mechanism induce processes ultimately increasing the telomeric DNA content. Experimental evidence indicates that it occurs in a telomerase-independent manner, presumably via the alternative lengthening of telomeres (ALT) mechanism. The third part of this research cycle (Ruzanova et al., 2025) characterizes the telomere structure and function, as well as the molecular mechanisms responsible for maintaining chromosomal telomere length.

Briefly, telomeres are genomic regions at the ends of linear chromosomes. In vertebrates, telomeric DNA consists of TTAGGG repeats bound by specialized proteins that form telomeric heterochromatin, thus modulating biological functions of telomeres. The terminal portion of single telomere strand, together with capping proteins, forms a complex protecting the recombinogenic structure against recognition by factors initiating the DNA damage repair mechanism. It is commonly believed that because of the conserved replication mechanism, DNA polymerase cannot completely replicate linear DNA templates, leading to progressive telomere shortening (Harley et al., 1990). Critically short telomeres cannot bind a sufficient amount of defense proteins, thus exposing double-stranded ends. This highly recombinogenic structure triggers the DNA damage repair mechanism, which in turn activates cyclin-dependent kinase inhibitors p21 and p16 and arrests proliferation (Stein et al., 1999). Despite the critical shortening, these telomeres preserve a certain amount of proteins, which prevents fusion of telomeres in different chromosomes but does not halt the activated repair mechanism. Proliferative arrest becomes permanent in this situation. Cellular senescence, the primary trigger of organismal involution and numerous age-related diseases, is initiated and sustained (He, Sharpless, 2017). Telomere-associated repair foci arising from attacks of telomeric replication forks by the activated molecular repair machinery are among the senescence markers.

It was demonstrated that DNA repair foci associated with incurred damage also arise within telomeric heterochromatin at long telomeres in terminally differentiated cells (Di Micco et al., 2021). In this case, the mechanism initiating cellular senescence in non-dividing cells can be conceptualized as follows: in proliferating cells, telomerebinding proteins inhibit DNA repair in cis, thereby preventing chromosomal fusion. Consequently, the insensitivity of dividing cells to DNA damage repair within telomeric heterochromatin in shortening telomeres conflicts with the molecular repair machinery and the overall repair process. This conflict induces intercellular propagation of signals from the DNA damage response mechanism and leads to formation of repair foci within telomeric heterochromatin of long telomeres in terminally differentiated cells. Instead of being repaired, the repair foci formed in telomeres of these cells are accumulated and induce an aging-like phenotype. Following this logic, persistent activation of the DNA damage repair mechanism is a common causal event that underlies replicative cellular senescence driven by critically short telomeres as well as aging-like states induced by damaged telomeres in non-dividing cells (Rossiello et al., 2022).

Apoptosis or autophagy is induced in the case of severe telomere dysfunction, being accompanied by all the associated molecular events (Nassour et al., 2019). Senescent cells acquire a senescence-associated secretory phenotype, characterized by secretion of a set of pro-inflammatory cytokines and negatively affecting the extracellular matrix structure and viability of stem cells (Tchkonia et al., 2013).

Hence, the inability to restore the lost telomeric heterochromatin content is among the causal events behind cellular senescence and its propagation throughout the organism. The conflict arising from insensitivity to telomeric DNA damage repair mechanisms further underscores the predominant role of chromatin DNA integrity in initiation of aging. Activation of the DNA damage repair mechanism and accumulation of telomeric repair foci are also presumed to be associated with other aging-related processes such as mitochondrial dysfunction, altered nutrient sensing, impaired autophagy, loss of proteostasis, and epigenetic dysregulation. In this regard, a “telomere-centric” rationale has been proposed for explaining many features of aging (Chakravarti et al., 2021), where telomere shortening being considered a key characteristic. The numerous agingassociated diseases are linked to telomeric heterochromatin attrition (Rossiello et al., 2022).

(Chakravarti et al., 2021), where telomere shortening being considered a key characteristic. The numerous agingassociated diseases are linked to telomeric heterochromatin attrition (Rossiello et al., 2022).

Our work, under the “telomere-centric” concept of aging and its propagation to hematopoietic progenitors, proposes an experimental approach to assess the relationship between the lifespan of experimental animals and telomeric DNA content in HSCs and HSC descendants. The experimental design of the study involved reinfusion of a suspension of bone marrow cells treated ex vivo with preparations of fragmented genomic DNA (hDNAgr), angiogenin, and two inducers administered simultaneously, into aged animals. Recombinant human angiogenin was selected as a comparator because its application had also been shown to increase telomeric DNA content in HSC descendant cells; however, unlike the DNA preparation, this rise was associated with activation of telomerase gene expression (Ruzanova et al., 2025). These treatments were expected to extend the lifespan of the experimental animals.

## Materials and methods

**Experimental animals. **Sexually mature male Wistar rats (weight, 400–450 g) were used for the pilot experiments. At experiment initiation, the rats were aged 13.5 months. Eighteen inbred female mice (weight, 50–60 g) were also included in the study; they were aged 12 months at experiment initiation. The animals were procured from the breeding facility of the Research Center for Biomedical Technologies, Federal Medical and Biological Agency of the Russian Federation, and had valid veterinary certificates. Following a two-week quarantine period, the animals were housed in the vivarium of the A.F. Tsyb Medical Radiological Research Center until they reached the appropriate age for starting the experiment. The animals were housed in polypropylene cages (five animals per cage) and had ad libitum access to food and water. The manipulations involving laboratory animals were conducted in accordance with the State Standard GOST 33044-2014 “Principles of Good Laboratory Practice”. Euthanasia was performed under ether anesthesia followed by cervical dislocation.

Male CBA/Lac mice aged 14 months and female Wistar rats aged 16 months, bred at the Vivarium of Conventional Animals core facility of the Institute of Cytology and Genetics SB RAS (Novosibirsk, Russia), were used for the repeated experiments. The animals were housed in groups of 6–10 mice and 3–4 rats per cage, with ad libitum access to food and water. All animal experiments were approved by the Animal Care and Use Committee of the Institute of Cytology and Genetics SB RAS. Mice were euthanized by cervical dislocation; rats were euthanized by CO2 inhalation or decapitation. Bone marrow cells were isolated from 14-month-old and 2-month-old male CBA/Lac mice, as well as 15-month-old male and a 2.5-month-old female Wistar rats.

**Isolation of bone marrow cells.** For isolating bone marrow, mice were euthanized; femurs and tibias were removed; epiphyses were separated; the medullary cavity was washed with DMEM+2 % FBS. The cell suspension was passed through a 21 gauge needle several times to remove rosette-forming cells and filtered through a 40 μm mesh. The cells were pelleted by 10-min centrifugation at 400g and resuspended in a buffer containing 130 mM ammonium chloride for erythrocyte lysis during 3–5 min. Subsequently, the buffer was diluted tenfold with PBS, and the cells were centrifuged again. The resulting cell pellet was resuspended in DMEM medium, and cells were counted in a Goryaev chamber.

**Treatment of bone marrow cells with inducers. **In the pilot experiment, bone marrow cells isolated from animals were incubated in the presence of hDNAgr for 1 h at room temperature (20–22 °C). A total of 200 ng of hDNAgr was used for treating 1×106 bone marrow cells.

In the repeated experiment, bone marrow cells isolated from animals were incubated in the presence of inducers for 1 h in an atmosphere of 5 % CO2 (95 % humidity, 37 °C): 500 μg hDNAgr, or 500 ng of angiogenin, or 500 μg hDNAgr + 500 ng of angiogenin per 3×106 cells in 1 mL of serum-free DMEM.

**hDNAgr preparation.** Human genome DNA reconstructor (hDNAgr) was isolated from placenta of healthy females. DNA was ultrasonically fragmented to 1–20 nucleosome monomers (200–2,000 bp), deproteinated using proteinase K, and isolated by phenol-chloroform extraction.

**Angiogenin.** Angiogenin was provided by Angiopharm Laboratory LLC (Novosibirsk, Russia). Angiogenin was labeled with Cy5 according to the manufacturer’s protocol (Lumiprobe, Germany).

**Intravenous administration of a bone marrow cell preparation.** In the pilot experiment, the experimental mice and rats received a single dose of 1×106 bone marrow cells in 0.3 or 0.5 mL of 0.9 % sodium chloride solution administered into the tail vein. Control animals intravenously received 0.3 and 0.5 mL of 0.9 % sodium chloride solution, respectively.

In the repeated experiment, mice and rats were reinfused a single dose of 1×106 bone marrow cells in 0.2 or 0.5 mL of 0.9 % sodium chloride solution into the tail vein. Control animals were reinfused with 1×106 untreated bone marrow cells isolated from old and young animals.

**Assessment of the effect of bone marrow cell preparation.** Animals were examined daily throughout the entire study. General behavior and health status of the animals were monitored. External morphology was assessed and documented through photographs. Animals’ body weight was measured. The morphology of spontaneous tumors was analyzed, and natural mortality among the experimental animals was documented.

**Postmortem analysis of mouse organs. **Organs and tumors were isolated from the animals and fixed in 4 % neutral paraformaldehyde. Samples of organs were dehydrated using increasing concentrations of ethanol, cleared in xylene, and embedded in paraffin. Paraffin sections up to 5 μm thick were stained with hematoxylin and eosin. Visualization and microphotography of the specimens were performed using an Axio Imager Z1 light microscope (Carl Zeiss Microscopy, Germany).

**Preparation of blood smears.** Blood smears were prepared using blood collected from the tail vein. The smears were fixed in methanol (OJSC Vekton, Russia) for 6–10 min, rinsed with water, dried, and stained using the Romanowsky–Giemsa protocol at pH 7.4. The prepared slides were examined under a Leica DV 4000V microscope (Germany) using transmitted light with immersion oil at a magnification of ×100.

**Preparation of bone marrow smears.** Bone marrow smears were prepared either by the conventional smear preparation procedure using a small amount of bone marrow cell suspension or by imprinting of animals’ thoracic bone sections. Further preparation of bone marrow specimens was conducted using the same procedure as the one employed for blood smear preparation

**Analysis of changes in telomeric DNA content.** Quantification of telomeric DNA content was carried out using bone marrow cells isolated from inducer-treated animals 12 months after reinfusion, as well as human bone marrow cells cultured for 15 days on methylcellulose after exposure to inducers. The bone marrow cells were embedded into 1 % low-melting-point agarose blocks (5×105 cells per block). Prior to analysis, the agarose blocks were stored in 0.5 M EDTA at 4 °C. Before electrophoresis, the blocks were rinsed in TE buffer and incubated in the presence of a lysis buffer (50 mM EDTA, 1 % sarkosyl (Serva, Germany), and 1 mg/mL proteinase K (Thermo Fisher Scientific, USA)) for 20 min at 50 °C. Next, the low-melting-point agarose blocks were secured in the wells of agarose block and subjected to electrophoretic separation using pulsed-field gel electrophoresis in the following mode: 3 s forward pulse; 1 s reverse pulse; RAM factor, 0.9.

Next, DNA was transferred onto a Hybond N membrane using the capillary transfer method in 20×SSC (Maniatis et al., 1984). DNA samples were UV-annealed to the membrane for 10 min and stored until hybridization.

The membrane with the crosslinked DNA was placed into 50 mL of prehybridization buffer containing 0.1 % SDS, 5×SSC, 5×Denhardt’s solution, and 100 μg/mL total yeast RNA, and incubated at 37 °C for 1–3 h. The labeled DNA specimen (32P-labeled oligonucleotide G-probe – (TTAGGG)9; C-probe – (CCCTAA)9) was denatured by 10-min boiling and added to 50 mL of hybridization buffer containing 0.1 % SDS, 5×SSC, 5 % dextran sulfate 500,000, and 100 μg/mL total yeast RNA. The prehybridization solution was removed, and hybridization buffer containing the labeled probe was added to the membrane after mixing. Hybridization was conducted overnight at 37 °C under permanent stirring. After hybridization, the membrane was washed thrice (15 min each washing cycle) with a solution containing 0.1 % SDS and 0.1×SSC at 37 °C. The hybridization conditions (buffer system, temperature, and number of washing cycles) for short oligonucleotides were optimized empirically based on multiple experiments with radioactive phosphorus; the temperature typically ranged from 37 to 42 °C (Dolgova et al., 2012).

The membrane with the transferred specimens was exposed to a K-type screen. Radioisotope specimens were scanned using a PharosFX system. Images were analyzed using the Quantity One software by measuring spot density (intensity/mm2) or using the GEL-Pro software.

**Statistical analysis **was conducted using the Statistica 8 (StatSoft, USA) and GraphPad Prism 8.0.1 software (GraphPad, USA). Survival analysis was performed by constructing Kaplan–Meier curves using the log-rank (Mantel– Cox) test. The significance of differences was assessed using the Mann–Whitney U-test. The observed differences were considered statistically significant at p < 0.05.

## Results

**The effect of reinfusion of hDNAgr-treated bone marrow cells (HSCs) on the lifespan and general condition of experimental animals**

Using the model characterizing changes in telomeric DNA content and the FISH data, we demonstrated that genetic information contained in extracellular DNA fragments can be incorporated into the recipient genome, in either an integrated, in complex with chromatin or a circular form. The findings attest to an increase in telomeric DNA content and the emergence of numerous sites on chromosomes hybridizing with DNA material that originally had an extrachromosomal localization (as evidenced experimentally they can be represented by circular structures encompassing chromosomal DNA strand or they form a complex with chromatin and coexisting in these forms for a certain period (Ruzanova et al., 2025)).

The telomeric DNA content is known to be a biomarker of lifespan (Rossiello et al., 2022). A series of experiments evaluated the lifespan of experimental mice and rats. Xenogeneic human DNA was employed, which was consistently increasing telomeric DNA content in mouse and rat cells during cloning experiments, suggesting that there were conditions conducive to lifespan extension in these animals. However, we were acutely aware that, along with telomere elongation, there can occur an uncontrolled DNAlevel interplay between human DNA fragments and rodent chromosomes, potentially adversely affecting condition of the experimental animals. Moreover, our studies, including the present work, revealed that only approximately 1 % of the genome can be delivered into the cell. It implies that internalization of this DNA will be highly degenerate. Different genomic DNA will enter different cells, further exacerbating the potential sequelae of the uncontrolled interplay between xenogeneic extracellular human DNA and rodent chromosomal DNA. Collectively, these considerations suggest that such treatment is inherently unpredictable and may result in either the anticipated extension of animals’ lifespan or an opposite unfavorable outcome.

*The effect of reinfusion of inducer-treated bone marrow cells (HSCs) on the lifespan and general condition of mice*

For conducting the pilot study of the effect of reinfusion of hDNAgr-treated bone marrow cells (HSCs) on the lifespan of animals, we selected nine outbred female mice of identical weight, without manifestations of spontaneous subcutaneous tumors. Five mice in the control group were intravenously administered with 0.9 % sodium chloride solution; the study group comprising four mice were infused with hDNAgr-treated bone marrow cells. The age of mice at experiment initiation was 12 months. The mean body weight of the experimental and control mice was 42.1 ± 2.4 g.

**Fig. 1.**
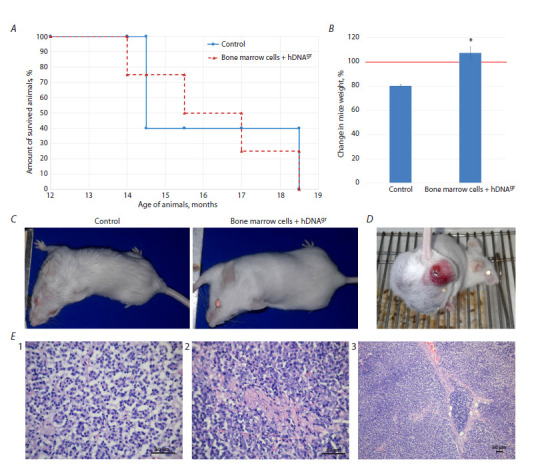
The effect of hDNAgr on the lifespan of mice. A – the survival rate of animals; B – changes in body weight in 14-month mice in the control and experimental groups compared to the baseline weight at an age of 12 months at experiment initiation point taken as 100 % (shown with a red line). * Statistically significant differences compared to the control group, p < 0.05; Mann–Whitney U-test. C – comparative external appearance of animals in the control and experimental groups at an age of 16 months; D – external appearance of a tumor-bearing mouse in the experimental group at an age of 16 months; E – histopathological analysis of the tumor in a mouse in the experimental group at an age of 16 months: 1 – the histostructure of mouse mammary carcinoma. ×40 magnification. 2 – micronecrosis within the carcinoma parenchyma. ×40 magnification. 3 – connective tissue septum within the carcinoma parenchyma. ×10 magnification. Hematoxylin and eosin staining.

Figure 1A shows the dynamics of death among experimental animals. The mean lifespan of mice was approximately 16 months: 487 ± 30 days in the control group and 490 ± 31 days in the experimental group. Two months after experiment initiation, body weight gain was observed for mice in the experimental group, whereas control mice experienced a mean weight loss of ~ 20 % (Fig. 1B). The physical condition of mice in the experimental group significantly differed from that in the control group (Fig. 1C). Control mice were less active during the terminal stage of life, had a ruffled hair coat and partial absence of undercoat hair. In contrast, mice reinfused with hDNAgr-treated bone marrow cells maintained activity and normal feeding behavior, they had a smooth hair coat with well-defined underfur hair.

In the experimental group of mice, one animal aged 16 months was found to have a neoplasm: a spontaneously developing tumor nodule in the pelvic region (Fig. 1D). The tumor was rapidly growing and, two weeks after its detection, reached a volume of 7.5 cm3. The tumor-bearing animal was withdrawn from the experiment for macroscopic and histopathological examination

The conducted histopathological analysis revealed that the mouse tumor parenchyma consisted of cuboidal epithelial cells with basophilic cytoplasm, which contained moderately polymorphic nuclei occupying approximately half of the cells and well-defined nucleoli. Glandular epithelium tending to form acinar or papillary structures was detected in some areas (Fig. 1E1). Mitotic figures and apoptotic bodies were rare. Small number of micronecrotic regions was revealed (Fig. 1E2). Chaotically distributed epithelial cells were found; a subtle lobular pattern of tissue was observed in some regions, being confined by thin connective tissue septa containing small blood vessels, with erythrocytes present in them. The connective tissue septa were thickened in some places to acquire a protruding structure (Fig. 1E3). These findings suggested spontaneous development of mammary carcinoma.

**Fig. 2.**
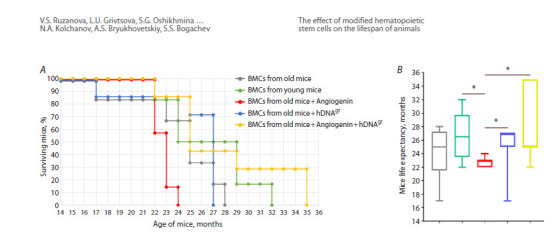
Analysis of lifespan of experimental mice in groups A – the Kaplan–Meier curve; B – lifespan of mice. * Statistically significant intergroup differences in the lifespan of mice, p < 0.05, log-rank (Mantel–Cox) test.

CBA/Lac mice were chosen to conduct the repeated experiment in mice. The animals were divided into five groups: (1) those reinfused with bone marrow cells from old animals; (2) those reinfused with bone marrow cells from young animals; (3) those reinfused with bone marrow cells from old animals treated with angiogenin; (4) those reinfused with bone marrow cells from old animals treated with hDNAgr; and (5) those reinfused with bone marrow cells from old animals treated with angiogenin + hDNAgr. Figure 2 shows an analysis of the lifespan of mice.

It was demonstrated that reinfusion of HSCs treated with hDNAgr had no effect on the lifespan of mice compared to those reinfused with bone marrow cells from old animals (Fig. 2B). Like in the first experiment, this finding suggests that treatment with hDNAgr has no effect on the lifespan of mice. Mice reinfused with angiogenin-treated bone marrow cells from old animals rapidly developed a symptom cluster eight months after treatment, resulting in weight loss (down to 17 g) and subsequent death within a short period (1 month) (Fig. 2).

The longest lifespan was observed in the groups of mice reinfused with bone marrow cells from young animals and from old animals treated with angiogenin + hDNAgr, with one and two mice surviving up to 32 and 35 months, respectively (17 and 30 % of the total number of mice). Four pivotal results of the study using the mouse model have been obtained

1. Treatment of bone marrow cells with xenogeneic dsDNA (hDNAgr) has no effect on the lifespan of mice. We attribute the reasons for that to the complex pleiotropic effects of fragments of exogenous heterologous dsDNA after its internalization into hematopoietic stem cells (HSCs) on the “fundamental metabolic constants responsible for aging of an organism”. Another fact important to consider is that a hematopoietic stem cell contains only 0.1–1.0 % of extracellular DNA, which constitutes a negligible portion of the genome. It means there is always a probability that telomeric repeat DNA is not delivered into the cell or its amount is insufficient for amplifying site-specific integration.

2. Reinfusion of bone marrow cells from mouse pups to old mice significantly increases the lifespan of mice in the group (up to 32 months).

3. Simultaneous administration of angiogenin and hDNAgr, internalized by hematopoietic stem cells (HSCs) (either by the same cell, or by different cells, or in a mixed manner), significantly extends the lifespan of mice (up to 35 months), outperforming the results obtained for mice reinfused with bone marrow cells from mouse pups. It indicates that two independent HSC activators, belonging to two different classes of polymers, have a synergistic effect and favorably influence the “fundamental metabolic constants” of HSCs responsible for cell aging, being accompanied by extension of the lifespan of experimental mice. The observed phenomena need to be further studied experimentally.

4. Angiogenin administered in the monotherapy mode induced a specific symptom cluster in mice, being the reason for death of mice in this group. Histopathological analysis was conducted to understand what events had occurred in the bodies of mice in response to angiogenin processed cells infusion (Supplementary Materials 1 and 2)1. One mouse had no pathological changes in the examined tissues and organs. In two other animals, areas of atypical tissue in the liver and kidneys were identified. The key pathological changes were most prominent in the liver and kidneys. Hepatocyte dystrophy was observed in the liver, being accompanied by polymorphonuclear infiltration, corresponding to the morphologic pattern of acute hepatitis. Significant dystrophic changes in the tubular and glomerular epithelium were observed in the kidneys, corresponding to acute kidney injury. Both types of destructive pathology affect vital excretory systems and could have been responsible for animal death related to angiogenin exposure. However, histopathological analysis of an animal reinfused with bone marrow cells from old animals showed nearly identical results. Atypical tissue was also detected in its liver and kidneys. Parenchymal inflammatory changes were detected in the liver, similar to those observed in angiogenin-treated mice. The kidneys also had dystrophic changes in tubules and glomeruli. This fact suggests that all these changes may result from aging of the animals. Similar changes were observed in the liver and kidney parenchyma in two other groups of animals (those reinfused with bone marrow cells from young animals and hDNAgr-treated cells), but there were no signs of inflammation. This fact also suggests that both the detected pathological destruction of the liver and kidneys and other unidentified factors are the reasons behind death following exposure to angiogenin (Supplementary Material 1).

Supplementary Materials are available in the online version of the paper: https://vavilov.elpub.ru/jour/manager/files/Suppl_Ruz_Engl_30_3.pdf


Simultaneously to histopathological analysis of mouse tissues and organs, we analyzed blood and bone marrow of animals withdrawn from the experiment at the premortem stage. Bone marrow cells were normal in all the experimental groups. Elevated lymphocyte counts in blood were observed in the groups of mice reinfused with bone marrow cells from old animals and angiogenin-treated cells. In the group of animals reinfused with bone marrow cells treated with angiogenin + hDNAgr, erythrocytes had a pathological morphology (burr cells), being indicative of functional disturbances leading to pathological alteration of erythrocyte shape (Supplementary Material 2). However, these alterations did not affect the lifespan of animals in this group.

*The effect of reinfusion of inducer-treated bone marrow cells (HSCs) on the lifespan of Wistar rats*

A total of 35 animals were used in the first pilot experiment (JSC Clinical Hospital “NeuroVita”, Moscow, Russia) to study the effect of hDNAgr on the lifespan of female Wistar rats. The animals were divided into three groups: bone marrow donors; control group consisting of 10 animals that received an intravenous infusion of 0.9 % sodium chloride solution; and the experimental group consisting of 19 animals that were reinfused with hDNAgr-treated bone marrow cells. At the time of experiment initiation, the rats were aged 13 months. The mean body weight of experimental and control rats was 460.0 ± 18.2 g.

**Fig. 3.**
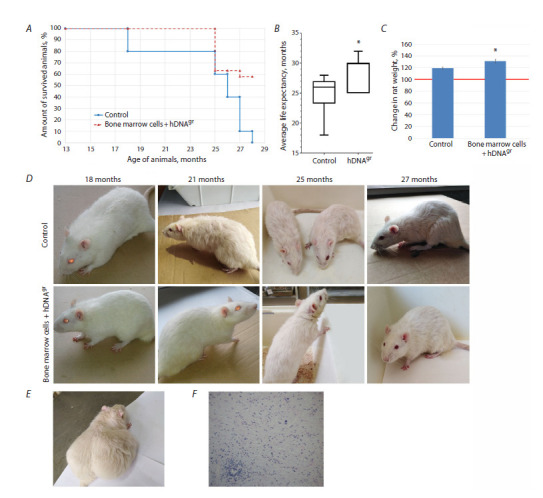
The effect of DNAgr on lifespan of Wistar rats. A – the survival rate of animals; B – average lifespan of the animals. * Statistically significant differences compared to the control group, p < 0.05; log-rank (Mantel–Cox) test. C – changes in the body weight of rats in the control and experimental groups aged 24 months compared to the baseline weight at an age of 13 months at experiment initiation point taken as 100 % (shown with a red line). * Statistically significant differences compared to the control group, p <0.05; Mann–Whitney U-test. D – comparative external appearance of animals in the control and experimental groups at different age; E – external appearance of a tumor-bearing rat in the experimental group at an age of 30 months; F – histopathological analysis of the tumor in a rat in the experimental group at an age of 30 months. Hematoxylin and eosin staining. ×20 magnification.

The survival dynamics of the experimental animals are shown in Figure 3A. The mean lifespan of the Wistar rat population in the control group was 717 ± 38 days or 24 months; in the group of animals that were reinfused with hDNAgr-treated bone marrow cells, it was 842 ± 25 days or 28 months, this difference being statistically significant (p <0.01) (Fig. 3B). At an age of 24 months, the mean body weight of rats in the experimental group was significantly higher than that in the control group (p < 0.05) (Fig. 3C).

Monitoring the physical condition of animals throughout the entire experiment revealed that already by the age of 18 months, the experimental rats significantly differed from those in the control group: they were characterized by better skin and fur condition and increased motor activity (Fig. 3D). Feeding activity in the experimental rats was higher than that in control animals. By the age of 21 months (eight months post-treatment), noticeable changes in physical condition became evident. Control rats were characterized by sparse fur, impaired grooming, and decreased muscle tone. In contrast, experimental rats at this stage were more active, displayed an exploratory behavior, maintained muscle tone, and had normal hair and skin. At an age of 25 months (12 months post-treatment), experimental rats retained mobility and exploratory behavior; muscle tone was slightly reduced, and movement coordination was undisturbed. Slight underfur hair loss was observed. Skin was clean; the rats exhibited no pathologic grooming behavior. Control rats at this age were less mobile, had reduced muscle tone, and partially impaired movement coordination. Significant hair loss and disturbances in grooming behavior were observed. A control rat that had reached the age of 27 months was characterized by decreased body weight, sparse fur, low activity, and impaired movement coordination. In contrast, 11 rats from the experimental group survived to this age. They had a slightly reduced body weight and sparse fur. However, the rats remained active, with preserved movement coordination, and exhibited an exploratory behavior.

We conducted a histopathological examination of internal organ tissues harvested from two rats following therapy with hDNAgr-treated bone marrow cells, which had been euthanized at an age of 30 and 32 months. A spontaneous neoplasm was detected in the abdominal cavity of the 30-month-old rat (Fig. 3E). Histopathological analysis of the upper pole of the right kidney revealed schwannoma, a benign tumor of neural origin, which appeared sporadically (Fig. 3E, F). The second rat in this group was euthanized at an age of 32 months. Histopathological analysis of the heart, lungs, liver, kidneys, spleen, brain, spinal cord, and red bone marrow revealed no signs of pathology.

**Fig. 4.**
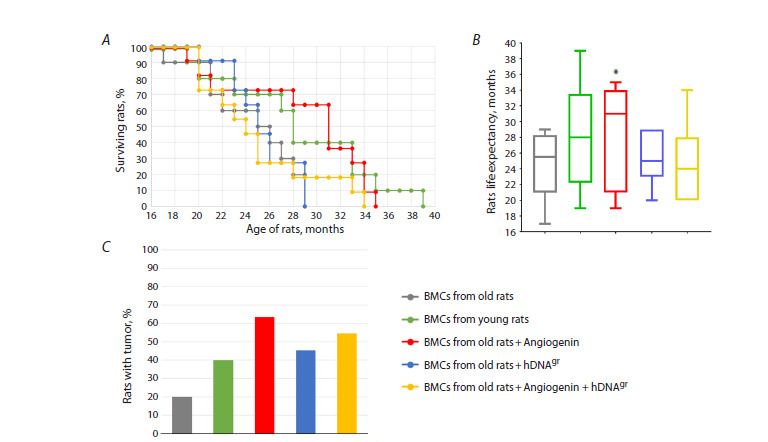
Analysis of the lifespan of experimental rats in study group. A – the Kaplan–Meier curve; B – lifespan of rats; C – the number of rats with tumors. * Statistically significant differences in the lifespan of rats compared to the control group, which received bone marrow cells from aged animals, p < 0.01, log-rank (Mantel-Cox) test.

A repeated experiment conducted using rats of the same line (Institute of Cytology and Genetics SB RAS, Novosibirsk, Russia) involved comparative assessment to evaluate the effects of reinfusing bone marrow cells (HSCs) from old animals, treated with angiogenin, hDNAgr, and angiogenin + hDNAgr. Control groups included rats treated with bone marrow cells from young and old animals (Fig. 4). In this study, we performed Southern blot analysis of changes in telomeric DNA content in the bone marrow 12 months after reinfusion of bone marrow cells from old and young rats and bone marrow cells treated with angiogenin, hDNAgr, and angiogenin + hDNAgr. Additionally, in this part of the study, we analyzed using rat and human models which telomeric strand (the G or C one) was amplified.

The following conclusions were drawn from the experimental results: (1) Reinfusion of bone marrow cells from aged animals treated with hDNAgr had no effect on lifespan extension compared to the control group (untreated bone marrow cells from aged animals) (Fig. 4). The average lifespan was approximately 25 months. (2) Reinfusion of bone marrow cells from young animals, as well as cells treated with angiogenin and angiogenin + hDNAgr increased the lifespan of rats in these groups (Fig. 4A, B). In all the experimental groups, 40–60 % of animals developed spontaneous tumors (Fig. 4C). In control groups, this parameter was 20–40 %. The higher rate of tumor occurrence in groups of animals reinfused with cells treated with angiogenin and angiogenin + hDNAgr can be attributed to the increased lifespan, which allowed more animals to develop neoplasms. Supplementary Material 3 summarizes the results of histopathological examinations of tumors in rats from different experimental groups. The findings indicate that rats in all the groups developed either spontaneous solid epithelial tumors, or squamous cell carcinomas, or glandular tumors that were identified as mammary gland tumors in some cases. All the tumors exhibited a low proliferative activity. We hypothesize that the developed neoplasms are related to animal housing rather than to tumorigenesis induced by hematopoietic stem cells. HSCs can differentiate into blood cells, but not into epithelial cells, which have been spontaneously transformed to tumors. Nevertheless, those are alarming findings requiring meticulous verification.

Furthermore, a high rate of pneumonia cases related to housing conditions was observed, which generally weakens the interpretative value of the results. Still, the findings from these pioneering studies provide a more meaningful basis for outlining the next level of research objectives

*Analysis of certain details of the telomere structure and comparative analysis of telomeric DNA content in bone marrow cells of experimental rats and in the specimen of human bone marrow cells*

Certain structural details of telomeres in the bone marrow cells of experimental rats and in a specimen of human bone marrow cells were analyzed using specific probes labeled with 32P targeting the G- and C-strand telomeric “tails”. Additionally, a comparative analysis was performed for: (1) changes in telomeric DNA content in the bone marrow cells of rats reinfused with untreated bone marrow cells from old animals (control) and cells from old animals treated with hDNAgr, angiogenin, and angiogenin + hDNAgr, 12 months post-treatment; (2) changes in telomeric DNA content in human bone marrow cells in control samples and hDNAgr-treated samples after 15-day culturing on methylcellulose.

Two independent experiments were performed to assess the telomeric DNA content in cell samples derived from rats reinfused with bone marrow cells from old rats treated with hDNAgr (the first experiment) as well as angiogenin and angiogenin + hDNAgr (the second experiment). Human bone marrow cells were also analyzed in the first experiment under methodological conditions identical to those used in the rat experiment.

**Analysis of telomeric DNA content.** Analysis of telomeric DNA content in bone marrow cells from control group rats and rats reinfused with hDNAgr-treated bone marrow cells, 12 months post-treatment, and in specimens of human bone marrow cells (control and hDNAgr-treated ones) on day 15 of cell culturing on methylcellulose.

Literature data demonstrate that telomeres have a long G tail. It means that hybridization with probes targeting different strands would generate a stronger hybridization signal when using C-strand probes.

**Fig. 5.**
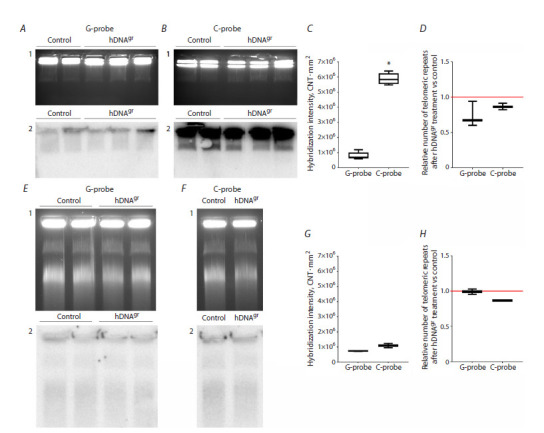
Analysis of certain details of the telomeric structure in bone marrow cells of experimental rats and in human bone marrow cell specimens: A–D – the rat model; E–H – the human model. A, B, E, and F – gel electrophoresis (1) and Southern blot analysis (2) of DNA samples after electrophoretic separation of lysed and deproteinized bone marrow cells embedded in low-melting-point agarose blocks. Hybridization was performed using 32P-labeled complementary oligonucleotides containing nine telomeric hexanucleotide repeats corresponding to G- and C-strand telomeric sequences (A, E – G probe; B, F – C probe); C, G – assessment of the total telomeric repeat content in the G- and C-strands in bone marrow cells in the control and experimental groups. The (CNT*mm2) values were compared using the GEL-Pro software. * Significant differences in hybridization intensity with the C probe with respect to hybridization intensity with the G probe, p <0.01, Mann–Whitney U-test. D, H – estimation of telomeric repeat content in hDNAgr-treated samples with respect to control ones. Relative values obtained by dividing the luminescence intensity of radioactive signals by the luminescence intensity of ethidium bromide for each lane, expressed in arbitrary units, were compared using the GEL-Pro software.

The results obtained for rats fully confirmed the available data (Fig. 5A–D). However, the efficiency of hybridization using different probes (G/C, with 32P-specific activity, and number of DNA hybridization probes being identical) did not differ significantly for human bone marrow cells. It implies that in the analyzed system, both strands are approximately of the same length. It also indicated that terminal reduction of telomeric heterochromatin had occurred, and telomeres had reached a critical length. We believe that it is most likely to be related to the patient’s disease (the cryopreserved bone marrow cells used had been harvested from a 59-year-old patient with multiple myeloma) (Fig. 5E–H).

In the same experiment, we compared the changes in telomeric DNA content between control and experimental cell samples for both models (Fig. 5D, H).

In rats reinfused with hDNAgr-treated bone marrow cells, no increase in telomeric DNA content (and telomere length) was observed 12 months post-reinfusion. These findings are consistent with the average lifespan data, which also showed no significant differences between the analyzed groups. They suggest that either hDNAgr did not induce telomere elongation in bone marrow cells in this particular experiment or such cells were eliminated during the 12-month follow-up period. Unfortunately, in the first pilot experiment, quantitative analysis using a radioactively labeled probe could not be performed because of some technical issues.

A similar result (no rise in telomeric DNA content) was obtained for the human model (Fig. 5H). We propose the following explanation in this case. For the control samples, amplification via the alternative lengthening of telomeres (ALT) mechanism requires freely accessible telomeric DNA rings to be present. These rings (t-circles) are formed after the detachment of a t-loop residing at the end of the long G tail in the telomere. According to the hybridization data, this long telomeric strand is absent in bone marrow cells derived from the analyzed sample, and t-circle formation is limited. This limitation explains why no difference in telomeric DNA content was observed in this particular experiment.

As mentioned previously, in the case of hDNAgr inducer, alternative lengthening of telomeres (ALT) is the main mechanism for telomere lengthening; telomeric DNA is amplified on t-circles formed by extracellular fragments via the rolling-circle mechanism. We believe that like for the mouse model, the lack of increase in telomeric DNA content in these particular experiments is related to ambiguity in the internalization process. It was demonstrated that only 0.1–1.0 % of extracellular DNA, a negligible portion of the genome, is internalized by a hematopoietic stem cell. It means that there will always be a risk that telomeric repeat DNA is not delivered into the cell or its amount is insufficient for amplification or site-specific integration. Some other explanations for this phenomenon, related to the biology of HSCs, are also possible; so further meticulous studies are needed (e. g., competitive elimination of a modified stem cells from the bone marrow by clones that have acquired clonal characteristics during age-related alterations).

Hence, in the first pilot experiment, hDNAgr-treated bone marrow cells reinfused to experimental animals had a significant effect on their quality of life during the terminal period, as well as increased their average lifespan. Evidence supporting the validity of the proposed concept was obtained in the experiment. Both anticipated biological effects were observed: a positive trend in the overall condition of aged animals throughout their remaining lifespan and a statistically significant extension of the lifespan of experimental rats.

In the repeated experiment, lifespan extension was observed in neither the mouse model nor in rats in the groups of hDNAgr-treated bone marrow cells. Moreover, molecular analysis revealed no changes in telomeric DNA content. In other words, these two parameters can be considered correlated.

As mentioned at the beginning of the article, the differences in the endpoints of two similar experiments can be attributed to the ambiguous behavior of the analyzed HSC system vs hDNAgr because of the potential uncontrolled interplay between xenogeneic extracellular human DNA and rodent chromosomal DNA.

**Fig. 6.**
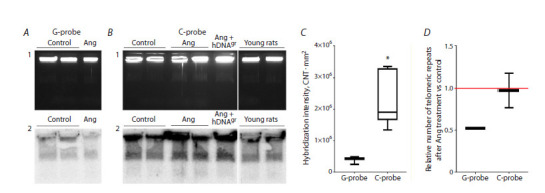
Analysis of certain details of the telomeric structure in bone marrow cells of experimental rats 12 months post-treatment. A, B – gel electrophoresis (1) and Southern blot analysis (2) of DNA samples after electrophoretic separation of lysed and deproteinized bone marrow cells embedded in low-melting-point agarose blocks. Hybridization was performed using 32P-labeled complementary oligonucleotides containing nine telomeric hexanucleotide repeats corresponding to G- and C-strand telomeric sequences (A – G probe; B – C probe)). C – assessment of the difference in the total content of G- and C-strand telomeric repeats in bone marrow cells in the control and experimental groups. The (CNT · mm2) values were compared using the GEL-Pro software. * Significant differences in hybridization intensity with the C probe with respect to hybridization intensity with the G probe, p <0.01, Mann–Whitney U-test. D – estimation of the relative number of telomeric repeats in angiogenin-treated samples with respect to control ones. Relative values obtained by dividing the luminescence intensity of radioactive signals by the luminescence intensity of ethidium bromide for each lane, expressed in arbitrary units, were compared using the GEL-Pro software.

In the second series of hybridization experiments, we compared the telomeric DNA contents in the cells of control rats and rats reinfused with bone marrow cells derived from old rats and treated with angiogenin alone and with angiogenin + hDNAgr 12 months post-treatment (Fig. 6). This analysis was needed because of the findings indicating that the lifespan of rats after treatment with angiogenin alone and in combination with hDNAgr was significantly longer than that of both control animals and animals treated with hDNAgr alone

A comparative analysis of luminescence intensity of the signals from ethidium bromide and radioactive labeling was conducted. Importantly, all the electrophoresis and hybridization conditions were identical in the experiment, making certain quantification possible. In all the samples, the G-strand telomeric repeats (C probe) were shown to dominate over the C-strand repeats (G probe) (the length of DNA probe and specific activity of both probes being virtually identical). This finding is consistent with the available literature data (Fig. 6A2, C).

Previous hybridization experiment demonstrated that treatment of cells from old rats with hDNAgr had no effect on the increase in telomeric DNA content in the bone marrow cells of experimental animals, and the average lifespan in this group did not differ from that in the control. Therefore, we have put forward a hypothesis that after treatment with angiogenin + hDNAgr, the main effect is caused by angiogenin per se rather than by hDNAgr, so the data for the “Angiogenin” and “Angiogenin + hDNAgr” groups can be pooled. The analysis revealed that the relative content of telomeric repeats for C- and G-strands in total DNA in rat groups did not significantly change compared to control. However, the content of G-strand repeats was significantly higher than that of C-strand repeats (Fig. 6C). The telomeric DNA hybridization data indicate that the substantial rise in the lifespan of rats following treatment with angiogenin alone or in combination with hDNAgr is not associated with an increase in telomeric DNA content (number of telomeric repeats), suggesting either that angiogenin does not activate endogenous telomerase activity (Ruzanova et al., 2025) or that other mechanisms are involved (e. g., competitive elimination of reinfused modified hematopoietic clones by bone marrow progenitors with clonal characteristics).

## Discussion

The efforts to extend longevity have been accompanying humanity throughout the entire history of civilization. Currently, there is a well-established understanding of biomarkers characteristic of aging and inherent to aged organisms, along with various anti-aging interventions being implemented (López-Otín et al., 2013, 2023; Proshkina et al., 2020; Zhu et al., 2021). Nonetheless, modern anti-aging approaches and diverse medicines developed relying on the insights into molecular processes occurring within cells and organisms, rarely enable outliving the coveted 100-year limit. In our view, it stems from the fact that aging is an involutionary integrative state of the organism with unknown causes of degradation rather than being merely a response of a specific functional system to lifespan that can be “corrected” using therapeutic procedures or interventions. In this context, maintaining a healthy lifestyle is at least half of the success in achieving a long life. The genetic factor also plays a predominant role in the development of anti-aging strategies, and current scientific efforts in gerontology primarily pursuit this aspect. These therapies include cellular reprogramming using Yamanaka factors (Takahashi, Yamanaka, 2006; Ocampo et al., 2016; Gowing et al., 2017; Brooks, Robbins, 2018; Sogabe et al., 2018), utilization of microRNAs that target multiple genes within networks regulating fundamental aging pathways (Vaiserman et al., 2016), combinatorial gene therapy (FGF21+αKloho+sTGFβR2) (Davidsohn et al., 2019), and telomere length extension therapies, primarily through activation of endogenous telomerase or transduction of the telomerase gene (Aubert, Lansdorp, 2008; Bernardes de Jesus et al., 2012; Li et al., 2017; Hong, Yun, 2019).

As previously mentioned, telomere length is a fundamental factor determining cellular aging and the development of many diseases of civilization, and in particular when telomere shortening events occur in stem cells (Rossiello et al., 2022). In this context, understanding approaches enabling telomere length extension with systemic organism-wide effects, rather than solely in cultured cells, poses a non-trivial challenge for both biology and clinical medicine. Almost all the identified telomere elongation methods involve targeting the telomerase complex and its regulatory genes (Aubert, Lansdorp, 2008). We failed to identify clinical strategies capable of increasing telomere length through an equally important alternative mechanism, alternative lengthening of telomeres (Lundblad, 2002; Hande, 2004; Pickett et al., 2009; Nabetani, Ishikawa, 2011; Rovatsos et al., 2011; Doksani, 2019; Loe et al., 2020).

Our study assessed the feasibility of extending the lifespan using the technology embedded in the novel concept of natural genome reconstruction, which is associated with the potential for in vivo telomere lengthening in hematopoietic stem cells.

Animal studies indicate that reinfusion of bone marrow cells treated with inducers is relatively safe. No prominent immediate pathological changes were observed in experimental animals across both models.

The findings regarding the lifespan of rats reinfused with bone marrow cells (HSCs) activated using fragmented human DNA suggest that there is a fundamental feasibility of extending the lifespan of aged animals. This effect is plastic: a statistically significant increase in lifespan was observed in some cases, whereas in other cases treatment had no effect on the lifespan of experimental animals.

In the cases when no effect was observed, the detectable telomeric DNA content in rat bone marrow cells 12 months post-treatment did not differ from that in the control samples. Clonal competition can be a putative mechanism explaining the absence of increased telomeric DNA content in bone marrow cells one year after treatment with hDNAgr. In this scenario, the rejuvenated clone with increased telomeric DNA content emerging after treatment as demonstrated in ref. (Ruzanova et al., 2025) could be outcompeted by dominant bone marrow residing clones that have acquired clonal characteristics. If this hypothesis is confirmed, it would be a crucial observation indicating that multiple initial interventions using modified bone marrow cells are needed to counteract the competitive expansion of dominant clones and achieve stable fixation of a trait in the bone marrow. Preliminary myeloablation is also possible, allowing reconstructed HSCs to be fixed in the vacated stromal niches.

Recombinant human angiogenin may exhibit systemic toxicity (a mouse model), which is mitigated when combining it with hDNAgr. The synergistic use of angiogenin and hDNAgr increases the lifespan in the group of mice.

The observed lifespan extension in rats after treatment with angiogenin is unrelated to telomere elongation, being determined by other properties of the factor, or clonal competition in the bone marrow, or a combination of both mechanisms.

**The concept of the “programmed clonal hematopoiesis” and “programmed death” mechanisms**

In their study, E.N. Proshkina et al. (2020) suggested aging to be a quasi-program, where the regulatory elements are not originally intended for its execution but are responsible for other processes such as cell growth, regeneration, stress response, and immunological protection. Therefore, it is inferred that the aging program does not exist, since there are no specific molecular or cellular subprograms that would assume the function of controlling the disintegration of the organism’s system elements with age. That’s exactly what it means, that the causes of aging are not understood yet, and it is only markers of aging that can be discussed.

Nevertheless, we have formulated a sufficiently adequate concept demonstrating that organismal aging is a program involving the dialectical unity of certain elements of its function and continuous interaction with the hostile environment, being related to the development of clonal hematopoiesis (this view is possibly characteristic of any stem cell system within the organism). This concept integrates all the revealed biological phenomena and related events. We also propose a method to overcome the sequelae of telomere shortening, fixation of pathological mutations, and their dominance in HSCs (as well as in stem cells of another origin), which could underlie future anti-aging therapy and treatment for diseases of civilization.

**The putative “programmed clonal hematopoiesis” and “programmed death” mechanisms. ** At birth, humans have two fundamentally self-regulating mutually related systems: HSCs (possibly all other types of stem cells involved in regeneration processes in the organism) and the entire population of somatic cells.

• HSCs are responsible for regeneration in the body. They are hidden in the most secluded location, the bone marrow. • Somatic cells interact with the hostile external environment.

The HSC population at birth is characterized by polyclonality, representing the full range of allelic variants required to perform regeneration function and maintain immune diversity.

The following features underlie the programmed clonal hematopoiesis and programmed aging mechanisms:

• HSCs internalize extracellular dsDNA fragments constituting approximately 0.02–1.0 % of the haploid genome. • During terminal differentiation, HSCs reorganize the chromatin architecture via induction of single-strand breaks and relaxation of chromosomal DNA strands, thereby inducing a “recombinogenic situation” during which dsDNA fragments located within internal cellular compartments and delivered into the cell under various circumstances can be integrated. • Having been internalized by a primitive hematopoietic cell, dsDNA fragments induce terminal differentiation of this cell. Hence, dsDNA fragments delivered into the cell both trigger a “recombinogenic situation” and participate in recombination process activated by them. • dsDNA fragments constantly circulating in blood plasma are remnants of apoptotic cells; their chromatin eventually hydrolyses to a size of 1–20 nucleosome monomers.

**The core of the mechanism. **After natural death via apoptosis and subsequent secondary necrosis, organismal cells, with the entire range of mutations accumulated over their lifespan, release their fragmented dsDNA harboring numerous genetic abnormalities within various genes into the bloodstream.

**Fig. 7.**
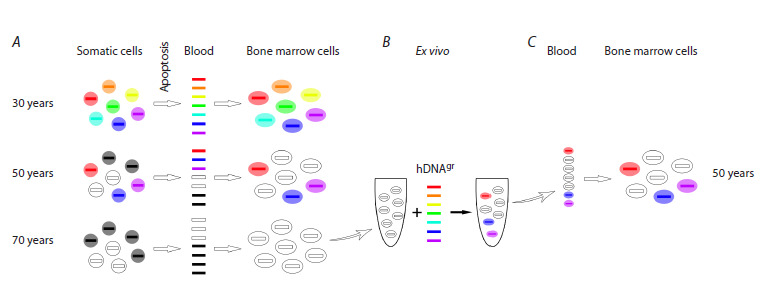
The mechanistic diagram. A – the emergence of mutations in somatic cells, their release into peripheral blood, and fixation within bone marrow cells. Formation of oligoclonal hematopoiesis; B – alterations in the HSC genome induced by fragments of extracellular dsDNA in the ex vivo system; C – reinfusion of cells with reconstructed genomes into the peripheral circulation and their fixation within the bone marrow. Recovery of polyclonal hematopoiesis.

This extracellular dsDNA is continuously transiently internalized by HSCs, where it induces commitment and is stochastically integrated into the genome, presumably at homologous regions, at specific time instants such as increased plasma DNA concentration following injury or ischemia. Gene conversion takes place, causing alterations in the genetics of HSCs: they lose their stem cell identity and acquire features characteristic of somatic cells that have responded to aggressive environmental stimuli. Over time, the HSC population becomes depleted as its pluripotent potential is exhausted, and the HSC system acquires oligoclonal features (Fig. 7).

This process reduces the DNA repair capacity of HSCs, and most importantly, results in loss of the original immune diversity, leading to physical aging of the organism.

It is possible to overcome the programmed clonal hematopoiesis and programmed aging mechanisms. It can be achieved by employing the same “weapon”: regularly providing HSCs with an extracellular dsDNA substrate derived from healthy young individuals, hDNAgr preparation being such a substrate. Healthy alleles will displace the accumulating mutant ones, and HSCs will maintain their pluripotent status.

## Conflict of interest

The authors declare no conflict of interest.
